# Low-Contrast BIC Metasurfaces with Quality Factors Exceeding 100,000

**DOI:** 10.1021/acs.nanolett.4c05880

**Published:** 2025-02-07

**Authors:** Keisuke Watanabe, Tadaaki Nagao, Masanobu Iwanaga

**Affiliations:** † International Center for Materials Nanoarchitectonics (MANA), National Institute for Materials Science (NIMS), 1-1 Namiki, Tsukuba, Ibaraki 305-0044, Japan; ‡ Department of Condensed Matter Physics, Graduate School of Science, Hokkaido University, Kita 10, Nishi 8, Kita-ku, Sapporo 060-0810, Japan; § Research Center for Electronic and Optical Materials, National Institute for Materials Science (NIMS), 1-1 Namiki, Tsukuba, Ibaraki 305-0044, Japan

**Keywords:** metasurfaces, bound states in the continuum, *Q* factors, all-dielectric, silicon, biosensors

## Abstract

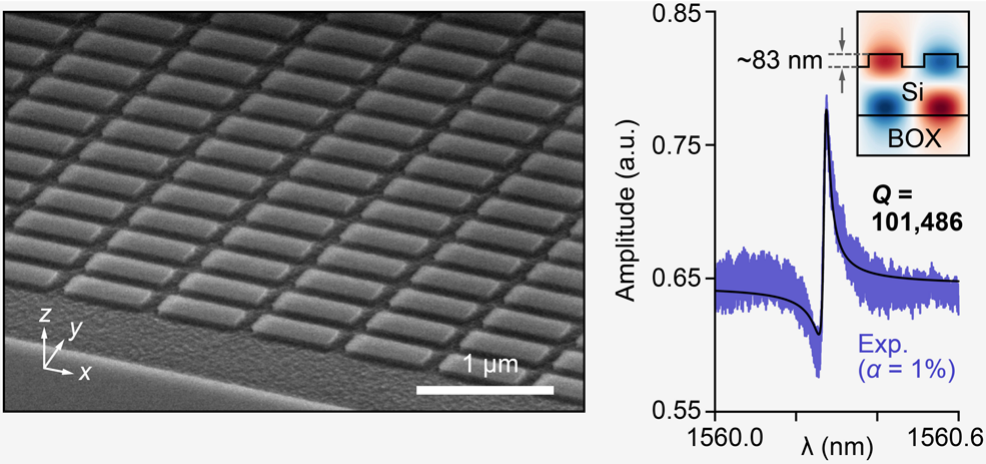

Dielectric metasurfaces operating at quasi-bound states in the continuum (qBICs) can achieve exceptionally high radiative quality (*Q*) factors by introducing small asymmetries into their unit cells. However, fabrication imperfections often impose major limitations on the experimentally observed *Q* factors. In this study, we experimentally demonstrate BIC metasurfaces with a *Q* factor of 101,000 under normal excitation of light in the telecom wavelength range achieved by employing low-contrast silicon pairs. Our findings show that such free-space accessible ultrahigh-*Q* factors can be attained by leveraging both the high radiative *Q* factors of higher-order qBIC modes and reduced scattering losses in shallow-etched designs. Additionally, we demonstrate stable sub-picometer-level wavelength fluctuations in water, with a limit of detection of 10^–5^ for environmental refractive index changes. The proposed approach can be extended to BIC metasurfaces with many other configurations and operating wavelengths for ultrahigh-*Q* applications in both fundamental physics and advanced devices.

Strong confinement and localization of light in nanofabricated structures are of great importance for diverse applications, including high-efficiency lasers,^1^ sensors,^2^ bioimaging,^3^ nonlinear enhancement,^4^ non-Hermitian optics,^5^ and topological photonics.^6^ The energy dissipation of confined light is quantified by the quality (*Q*) factor, and both the material selection and structural design play major roles for maximizing the experimental *Q* factors. To date, nanostructures with periodic arrays have been successfully employed to achieve high *Q* factors. Such nanostructure arrays include photonic crystals (PCs),^7^ plasmonic cavities,^8^ and metallic or dielectric metasurfaces that support surface lattice resonances^9,10^ and bound states in the continuum (BICs).^11^ Remarkably, BICs in dielectric materials have recently attracted considerable attention owing to their design flexibility in controlling radiative losses through precise structural design. A symmetry constraint dictates that symmetry-protected BICs are not accessible from free-space. However, true BICs can be transformed into quasi-BICs (qBICs) by breaking the symmetry of the unit cell, yielding finite radiative *Q* factors. This symmetry breaking enables the observation of sharp resonances under normal-incidence excitation. The high radiative *Q* factors and resonantly enhanced electric fields are the key features of the qBIC modes, offering novel strategies to enhance the functionality of optical devices.^12−14^ However, BIC metasurfaces, which often have a large ratio of nanostructure depth to in-plane dimensions, are susceptible to scattering losses owing to fabrication imperfections, thus limiting their experimental *Q* factors to the range of several hundreds to thousands. More specifically, the localized electric fields within high-index nanostructures overlap with the sidewalls, on which light scattering caused by surface roughness from nanofabrication drastically reduces the experimental *Q* factors. In other words, experimental *Q* factors cannot improve under large scattering losses, despite many researchers focusing on increasing the radiative *Q* factors obtained from numerical calculations. To resolve this problem, two approaches can be considered. The first approach involves utilizing imperfection-tolerant designs.^15,16^ Jin et al. proposed that merging off-Γ BICs with multiple topological charges into an isolated symmetry-protected BIC at the Γ point can preserve high *Q* factors over a broad range in the *k*-space, substantially suppressing out-of-plane scattering.^15^ This approach enabled the experimental demonstration of ultrahigh-*Q* factors up to 4.9 × 10^5^ in PC structures. However, topological charge engineering requires precise control in fabrication, because the merging BICs are extremely sensitive to structural parameters.^17^ Zhong et al. recently demonstrated toroidal dipole BIC metasurfaces with *Q* factors that are robust against variations in the shape of dimer nanoholes.^18^ The experimentally observed *Q* factors benefit from the fabrication tolerance of the toroidal dipole mode but remain limited to the order of 10^4^. The second approach focuses on imperfection-reduced designs. Huang et al. recently demonstrated a simple structure with a thin patterned photoresist layer on top of a silicon-on-insulator (SOI) wafer,^19^ achieving an ultrahigh-*Q* guided mode resonance with a *Q* factor as high as 2.4 × 10^5^ for PC structures. In this design, the absence of nanopatterning in the silicon layer minimized scattering losses typically caused by surface roughness from silicon etching. However, this ultrahigh-*Q* factor has not been demonstrated in BIC metasurfaces to date. More importantly, nanopatterned photoresist layers suffer from poor durability because they can be easily damaged, degraded, and dissolved in many solvents. Therefore, achieving monolithic high-*Q* metasurfaces with stable patterned layers remains challenging, which is essential for a wide range of optical device applications.

In this study, we propose and experimentally demonstrate a method to minimize fabrication imperfections in silicon metasurfaces by employing shallow-etched structures.^20−22^ Our approach increases the experimental *Q* factors by approximately 1 order of magnitude compared with conventional designs, pushing the limits of *Q* factors over 10^5^. We design, fabricate, and characterize BIC metasurfaces composed of arrays of silicon pairs with varying etching depths. Our results show that shallower etching simultaneously enhances the radiative *Q* factors and reduces scattering losses caused by fabrication imperfections in higher-order qBIC modes, leading to substantially improved experimental *Q* factors. Finally, we demonstrate highly stable refractometric sensing, achieving a limit of detection (LOD) at the 10^–5^ level using low-contrast BIC metasurfaces. We expect that the developed ultrahigh-*Q* silicon metasurfaces will find broad applications in fields requiring strong light–matter coupling at the nanoscale.

Figure 1a illustrates the proposed nanostructures, which are shallow-etched silicon pairs with etching depth *d*, fabricated on SOI wafers with a thickness of 400 nm. Silicon is chosen as the material for light confinement due to its high refractive index and transparency over a broad wavelength range. The buried oxide (BOX) layer is 2000 nm, which is thick enough to sufficiently suppress leakage losses to the bottom silicon substrate.^23^ The dimensions of the unit structure are period *P* = 760 nm, shallow rod length *L* = 610.8 nm, and shallow rod width *w* = 235.5 nm. The symmetry of the unit structure is broken by changing asymmetry parameter α = 2Δ*L*/*L*, which controls the radiation losses of the resonance mode emitted into the far-field. Figure 1b shows the simulated transmittance spectra for an infinite periodic structure computed using the finite-difference time-domain (FDTD) method (Ansys Lumerical). The resonance modes are excited by a normally incident *x*-polarized plane wave parallel to the major axis of the rectangular structures (see Supporting Information S1 for methods). For *d* = 0 (i.e., unpatterned silicon), the transmittance spectrum exhibits interference patterns owing to the multilayer configuration of the SOI wafer. When shallow-etched nanostructures with *d* = 82.7 nm and α = 0% (i.e., no asymmetry) are introduced, a large transmittance dip appears around the wavelength of 1870 nm caused by the destructive interference between the leaky guided-mode resonance and background continuum. When α = 5%, three BIC modes are transformed into qBIC modes. The cross-sectional *E*_*x*_ profiles shown in Figure 1c indicate that the metasurfaces support a fundamental mode (qBIC1) and higher-order modes (qBIC2 and qBIC3) in the wavelength range of interest (see Supporting Information S2). Figure 1d (left) presents the transmittance maps for different asymmetries α when *d* = 82.7 nm. The three qBIC modes retain narrow line widths even as α increases, suggesting that the low-contrast matesurfaces maintain large radiative *Q* factors across a broad range of α. Notably, the peak wavelengths of the three qBIC modes remain nearly constant as α changes because the overall volume of the shallow pair-rod remains unchanged despite changes in the lengths of the upper and lower rods. Figure 1d (right) shows the transmittance maps for different *d* when α = 5%. As *d* increases, the line widths gradually broaden, and the resonance wavelengths undergo a blueshift, as the modes penetrate more into surrounding air. Based on the calculation of field confinement factors in silicon *f*_Si_ (see Supporting Information S3), we find that the qBIC1 mode exhibits the highest *f*_Si_. However, the qBIC1 mode arises in the longer wavelength regime around 2.2 μm, necessitating smaller feature sizes to bring the resonance wavelength closer to 1.55 μm. This makes the qBIC1 mode more susceptible to fabrication imperfections. While the qBIC3 mode is close to the qBIC2 mode in wavelengths, its smaller *f*_Si_ is not conductive to achieving higher *Q* factors. Therefore, the qBIC2 mode emerges as the most suitable choice for the ultrahigh-*Q* factors, balancing a sufficiently large *f*_Si_ and a practical feature size.

**Figure 1:**
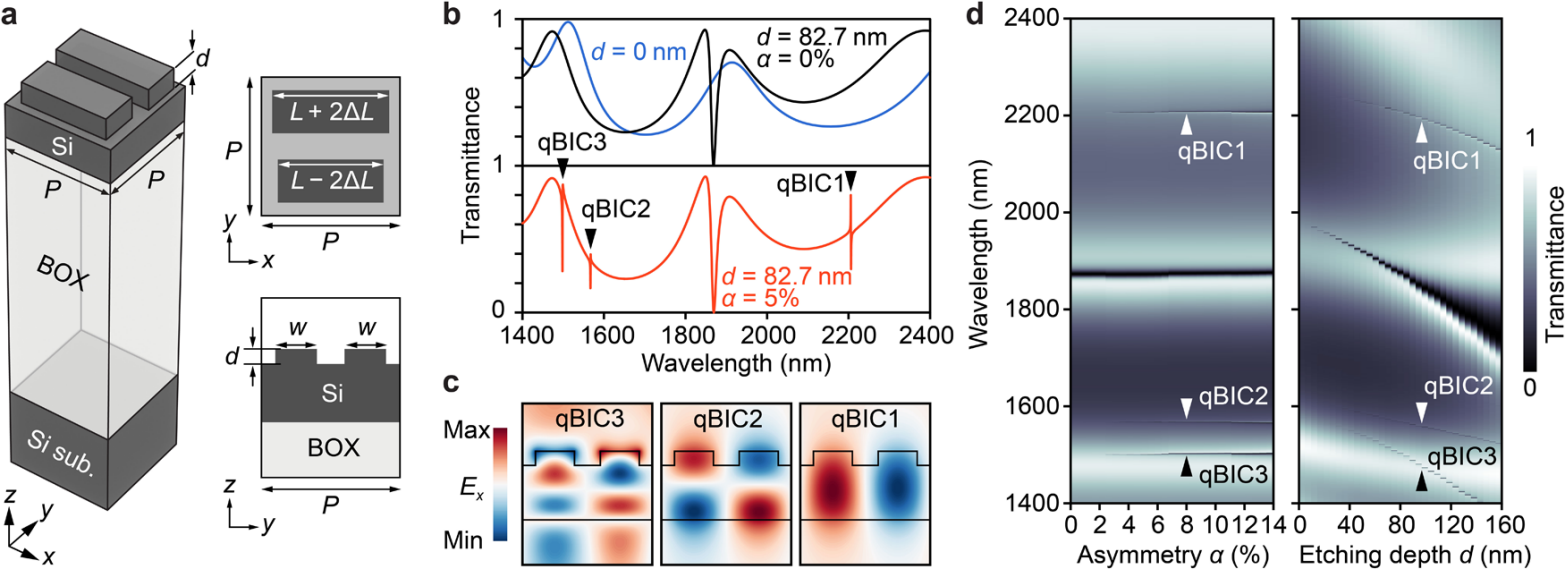
Low-contrast BIC metasurfaces fabricated on SOI wafers. (a) Schematic of structure and its dimensions. (b) Simulated spectra for unpatterned silicon (blue) and metasurfaces with *d* = 82.7 nm for α = 0% (black) and α = 5% (red). (c) Cross-sectional *E*_*x*_ distributions for three qBIC modes supported by proposed metasurfaces with *d* = 82.7 nm and α = 5%. (d) Simulated transmittance maps for different α when *d* = 82.7 nm (left) and for different *d* when α = 5% (right).

For the experimental realization of the designed low-contrast metasurfaces, electron beam lithography with a positive resist was employed, followed by dry etching using the Bosch process with SF_6_ and C_4_H_8_ gases, allowing the precise control of the silicon etching depth (Figure S1). Figure 2a, b presents scanning electron microscopy (SEM) images of the fabricated metasurfaces under highly controlled etching depth conditions. The fabricated device was characterized using a custom-built setup comprising a tunable laser and photodiode (Figure S2). Figure 2c shows the simulated and experimental transmittance spectra for *d* = 82.7 nm when normally incident *x*-polarized light was coupled with the metasurfaces with different α. The simulations and experiments were in good agreement, showing the qBIC2 resonance mode even for a small asymmetry α of 0.5%. Because the length difference between the upper and lower shallow rods for α = 0.5% is given by (*L* + 2Δ*L*)–(*L*–2Δ*L*) = 6.1 nm, the fabrication disorder is considered to be smaller than this value (see Supporting Information S4). As seen in the spectra, the resonance amplitude increased with increasing α, which is a typical behavior of qBIC modes. However, small sidebands occasionally appeared in the spectrum, possibly due to periodic size variations in the fabricated metasurfaces. These variations may arise from the writing order with regularity during EB lithography (see Supporting Information S5). Additionally, a slight blueshift in the experimental resonance wavelengths compared with the simulations was observed, which can be attributed to the rounded corners from lithography and/or the undercut geometry of sidewalls. Figure 2d shows a representative enlarged SEM image and the corresponding spectrum for α = 1%, showing a clear ultrasharp Fano resonance. The *Q* factor was extracted by fitting the transmittance spectrum with a Fano function, yielding a *Q* factor of approximately 101,000 at resonance peak wavelength of λ = 1560.3 nm.

**Figure 2:**
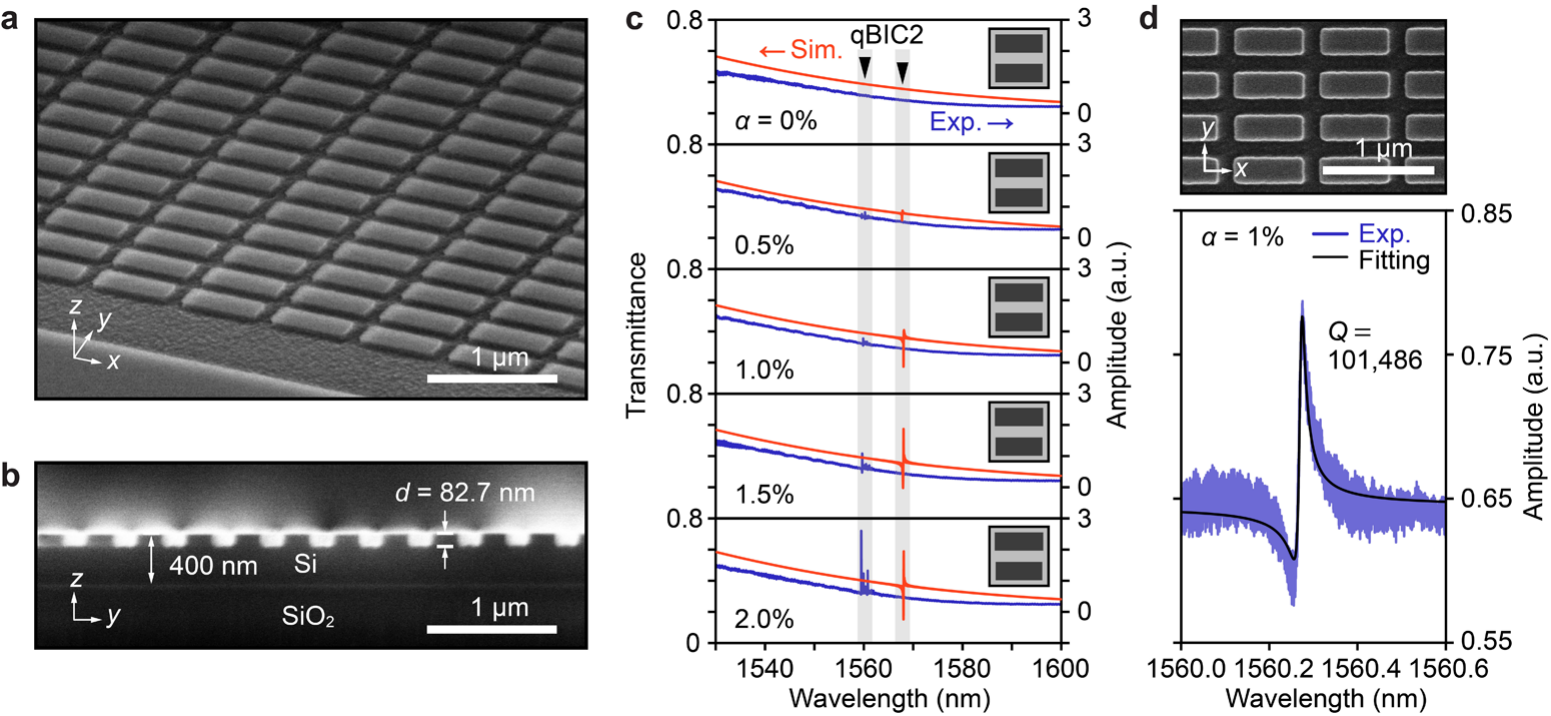
Fabricated low-contrast BIC metasurfaces with *d* = 82.7 nm. (a) Tilted and (b) *yz*-plane cross-sectional SEM images. (c) Simulated (red) and experimental (blue) transmittance spectra for different α. The gray regions indicate the wavelengths where the qBIC2 mode appears. Each inset shows a schematic of the unit structure with broken symmetry. (d) Representative SEM image (top) and transmittance spectrum (bottom) of a metasurface with α = 1%. The *Q* factor is extracted by fitting the transmittance spectrum with a Fano function.

To further elucidate the physics underlying the ultrahigh-*Q* factors, low-contrast BIC metasurfaces with etching depths *d* of 82.7, 116.1, and 149.5 nm were fabricated, and their *Q*-analysis was conducted by varying asymmetry α. First, we compared the simulated transmittance spectra for the metasurfaces with three etching depths, as shown in Figure 3a. As α increased, the resonance peaks blueshifted, and the resonance linewidths broadened, corresponding to an increased radiative component coupling into the external medium. The experimentally measured *Q* factors as a function of α are shown in Figure 3b. The black curves overlapping the experimental *Q* factors were fitted using *Q*^–1^ = *Q*_r_^–1^ + *Q*_scat_^–1^, where *Q*_scat_^–1^ represents the scattering losses arising from fabrication imperfections. *Q*_scat_ was determined using nonlinear least-squares curve-fitting method and assuming that *Q*_scat_ was independent of α (see Supporting Information S6). The loss component that does not contribute to far-field coupling is expressed as the sum of the material absorption and scattering losses owing to fabrication imperfections. However, in this study, we only considered scattering losses because the silicon and BOX layers exhibit negligible absorption in the wavelength range of interest. *Q*_r_ denotes the radiative *Q* factor calculated from complex eigenfrequencies obtained from the finite element method (FEM) method (COMSOL). Here, *Q*_r_ follows the typical inverse square relation, *Q*_r_ = *Q*_0_α^–2^ for qBIC modes,^24^ with *Q*_0_ being a constant that depends on the metasurface design and mode radiation characteristics (Figure 3c). As shown in Figure 3b, for small α, the experimental *Q* factors largely deviated from the inverse square relation for all etching depths and approached a fix value determined by *Q*_scat_. These behaviors indicated that the experimental *Q* factors were limited by an inherent *Q*_scat_. The difference between the experimental *Q* factors and *Q*_r_ widened as *d* increased, indicating an increase in scattering losses (i.e., decrease in *Q*_scat_) for larger etching depths. Figure 3d shows the experimentally determined *Q*_scat_ as a function of *d*, demonstrating that metasurfaces with smaller *d* exhibited reduced scattering losses, thereby yielding higher experimental *Q* factors. Although the highest *Q* factor exceeding 10^5^ was achieved for *d* = 82.7 nm in our experiments, further reductions in *d* below 50 nm did not yield any additional increase in *Q*_scat_ (see Supporting Information S7). This suggests that once a certain *Q*_scat_ is reached, further improvements in *Q* factors are unlikely due to inevitable fabrication errors and structural disorder.

**Figure 3:**
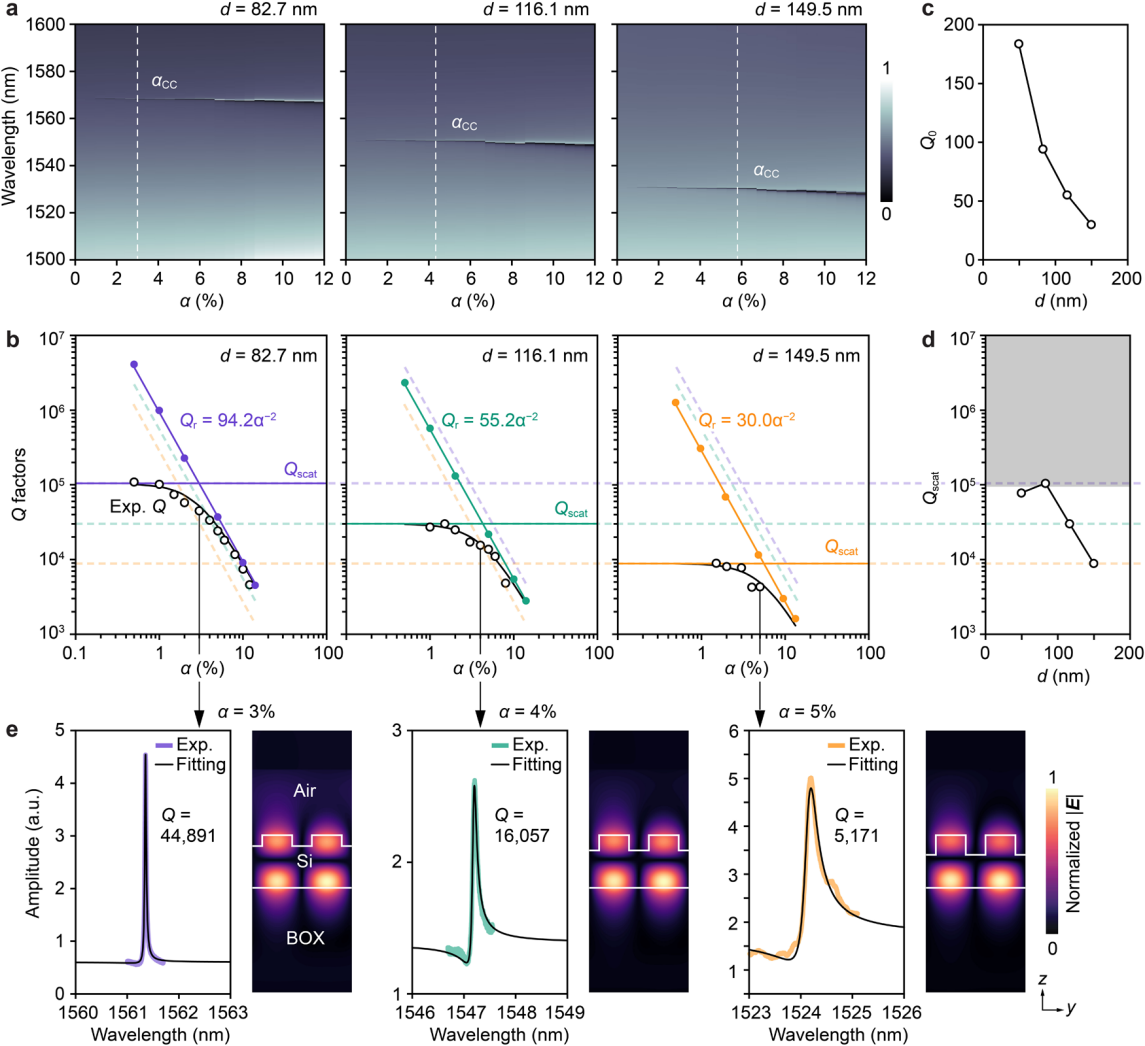
Characteristics of low-contrast BIC metasurfaces with different etching depths *d*. (a) Transmittance maps. The dashed lines indicate α values that satisfy the critical coupling conditions (α_CC_). (b) *Q*-analysis results. The radiative *Q* factors (*Q*_r_, filled circles) with their fitted lines and *Q*_scat_ obtained by fitting the experimental *Q* factors (black open circles) are shown. The *Q* factors were measured in air. (c) Coefficient *Q*_0_ obtained by fitting the inverse square relation of *Q*_r_. (d) Extracted *Q*_scat_. The gray region indicates the approximate upper limit of *Q*_scat_. (e) Experimental spectra and corresponding Fano fitting for metasurfaces near critical coupling conditions: α = 3% for *d* = 82.7 nm, α = 4% for *d* = 116.1 nm, and α = 5% for *d* = 149.5 nm. The normalized electric field |***E***| distributions for each condition are also shown.

As discussed below, the condition where *Q*_r_ = *Q*_scat_ corresponds to the critical coupling condition, which plays a crucial role in various applications such as sensing.^25−27^ In our experiment, we selected and measured metasurfaces with α = 3%, 4%, and 5% for etching depths *d* = 82.7, 116.1, and 149.5 nm, respectively, as these structural conditions were close to the critical coupling condition (α_cc_). The representative spectra are shown in Figure 3e. As expected, the experimental *Q* factors increased as *d* decreased. Figure 3e also shows the cross-sectional electric field distributions, indicating that the overlap between the localized electric fields and sidewalls of the shallow pair-rod structures reduces with decreasing *d*. This observation supports our conclusion that the increased *Q* factors for smaller *d* are attributable to the reduced ratio of nanostructure depth to in-plane dimensions, resulting in lower scattering losses due to nanostructured sidewall roughness. Considering that *Q*_0_ is larger for smaller *d*, we can conclude that the experimental *Q* factors increased by both the high *Q*_r_ and *Q*_scat_ values for the low-contrast metasurfaces.

Table 1 compares the ultrahigh-*Q* factors achieved in this study with previously reported experimental *Q* factors for free-space accessible all-dielectric nanostructures. While some reports demonstrate *Q* factors exceeding those achieved in this work, they rely on topological charge engineering and precise control of the incident angles,^15,28^ or suffer from poor durability of nanopatterned photoresist.^19,29^ In contrast, our low-contrast BIC metasurfaces supporting symmetry-protected BICs offer potential advantages in simpler fabrication and exhibit an experimental *Q* factor that is an order of magnitude higher than the highest *Q* factor reported to date. Given that most reported *Q* factors were 1000 or less,^14,30−32^ the *Q* factor of our metasurface was one or two orders of magnitude higher than typical values.

**Table 1:** Experimentally Observed Free-Space Accessible *Q* Factors Reported to Date in All-Dielectric Nanostructures^a^

Unit structure	Resonance type	Material	θ_inc_ (deg)	λ (nm)	Exp. *Q*	Ref.
Circular hole	GMR	Si_3_N_4_ on SiO_2_	0.2	490	32,000	(33)
Circular hole	GMR	Resist on SOI	0	1551	239,000	(19)
Square hole	GMR	Resist on Si_3_N_4_	0	779	1,100,000	(29)
Circular hole	Merging-BIC	Si slab	1.2	1568	490,000	(15)
Circular hole	Mini-BIC	Si slab	5.4	1573	1,090,000	(28)
Tilted bars	Chiral-BIC	TiO_2_ on SiO_2_	0	612	1250	(34)
Cuboid	Accidental-BIC	SOI	0	1538	5305	(35)
Square nanodisk with hole	TD-BIC	SOI	0	1497	4990	(36)
Nanodisk dimer	TD-BIC	SOI	0	1480	3142	(37)
Nanohole dimer	TD-BIC	SOI	0	1518	22,633	(18)
Nanodisk	SLR	a-Si on silica	0	1183	2750	(38)
Cylinder	SP-BIC	SOI	0	1425	1946	(39)
T-shape block	SP-BIC	Si on quartz	0	1588	18,511	(40)
U-shape block	SP-BIC	Si on sapphire	0	1548	3534	(41)
Block with nanogaps	SP-BIC	Si on quartz	0	1553	1233	(42)
Shallow tetramer	SP-BIC	Si_3_N_4_ on quartz	0	828	6061	(22)
Nanorod	SP-BIC	a-Si on fused silica	0	1505	4130	(43)
Double holes	SP-BIC	SOI	0	1553	36,964	(44)
Square nanopillar	SP-BIC	SOI	0	1685	2476	(45)
**Shallow pair-rod**	**SP-BIC**	**SOI**	**0**	**1560**	**101,000**	**This study**

a SP, symmetry-protected; TD, toroidal dipole; a-Si, amorphous silicon; GMR, guided-mode resonance; SLR, surface lattice resonance.

Next, we characterized the sensing properties of the low-contrast BIC metasurfaces. Previous studies have reported that the lowest LOD for environmental refractive index changes can be achieved under critical coupling conditions.^27,46^ Therefore, we compared the refractometric sensing performance of metasurfaces near the critical coupling conditions for different etching depths *d*. Figure 4a shows the experimental results, where aqueous solutions with different bulk refractive indices, adjusted by mixing isopropyl alcohol (IPA) and heavy water (D_2_O), were introduced into a polydimethylsiloxane (PDMS) microfluidic channel. The resonance peak wavelengths were recorded in real-time (see Supporting Information S8 for the original data). Here, we used D_2_O instead of H_2_O to avoid absorption loss of water in the wavelength range of interest, thus simplifying the analysis of sensing performance. As shown in the figure, the resonance peak wavelengths redshifted with increasing refractive index of the solution. Figure 4b shows the relation between the resonance peak wavelength shift Δλ and refractive index change Δ*n*. The environmental refractive index sensitivity *S* was calculated from the slope and found to be approximately 26 nm/RIU, which was smaller than the typical *S* of several hundred of nm/RIU for photonic sensors.^47−51^ Although *S* increased slightly with increasing *d*, the increment was small, being consistent with the simulation results (see Supporting Information S9). This reduced sensitivity is also attributed to the intrinsically strong confinement of the higher-order qBIC2 mode, which has weaker mode overlap with the external medium. In fact, the qBIC2 mode in low-contrast metasurfaces exhibits a field confinement factor in water that is ten times smaller than that of the fully etched BIC metasurfaces (see Supporting Information S10). Figure 4c shows real-time measurements of peak wavelength fluctuations δλ (= 3σ, where σ is the standard deviation) in D_2_O over a period of approximately 1 min. The δλ increased slightly as linewidths widened with increasing *d*. Here, the short evaluation period (approximately 1 min) was chosen to minimize the effects of wavelength drift. The minimum δλ was 0.79 pm for the metasurface with *d* = 82.7 nm (α = 3%). Figure 4d-g compares the experimental *Q* factors, sensitivity *S*, figure-of-merit (FOM), and LOD for different *d*. The FOM defined as FOM = *S*/fwhm, where fwhm is the full width at half-maximum, reached a maximum of 825 when *d* = 82.7 nm (Figure 4f). Thanks to our ultranarrow linewidths, this FOM was among the highest reported for experimentally demonstrated all-dielectric metasurfaces, such as asymmetric double bars (FOM ∼200),^52^ nanogap-enhanced blocks (FOM = 239),^42^ and dual-rectangular pillars (FOM = 418).^53^ The LOD given by δλ/*S* slightly reduced with decreasing *d* (Figure 4e), yielding values of 3.00 × 10^–5^, 3.28 × 10^–5^, and 3.51 × 10^–5^ for *d* = 82.7, 116.1, and 149.5 nm, respectively. Although the wavelength fluctuations were small at the sub-picometer scale, sensitivity *S* was also small. Therefore, increasing the *Q* factors does not lead to substantial LOD improvements due to the general trade-off between the *Q* factor and sensitivity. Nevertheless, our ultrahigh-*Q* silicon metasurfaces hold great potential for applications that require detection of local perturbations, such as single-molecule detection at high concentrations, leveraging both their high *Q* factors and small mode volume.^54^ Although a rigorous quantification of the mode volume warrants further investigation, the *Q*/*V* ratio could potentially be optimized through geometric modifications, including adjustments to the number of unit structures. Moreover, the proposed metasurfaces offer substantial advantages, including simple measurements based on position-insensitive vertical excitation from free-space, broad wavelength tunability (see Supporting Information S11), and availability of well-established complementary metal–oxide–semiconductor compatible fabrication processes, facilitating the practical implementation of low-cost sensing systems.

**Figure 4:**
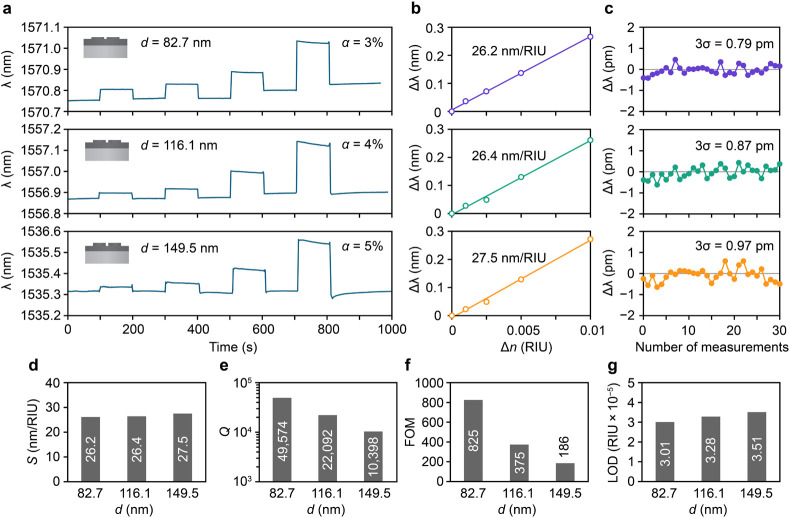
Refractometric sensing in low-contrast BIC metasurfaces with varying etching depths *d*. (a) Real-time measurement results. D_2_O solutions with different refractive indices were introduced sequentially, with refractive index variations of Δ*n* = 0.001, 0.0025, 0.005, and 0.01. After each step, the metasurfaces were rinsed with D_2_O. (b) Wavelength shift as a function of refractive index variation. Sensitivity *S* was derived from the curve slope. (c) Representative real-time fluctuations in resonance peak wavelength measured in D_2_O. (d)-(g) Comparison of *S*, *Q*, FOM, and LOD for different *d*.

In conclusion, we have experimentally demonstrated ultrahigh-*Q* factors exceeding 10^5^ in low-contrast silicon metasurfaces supporting higher-order qBIC modes. By designing the metasurfaces to minimize the overlap between the localized electric fields and sidewalls in shallow-etched nanostructures, we achieved high radiative *Q* factors while reducing scattering losses from fabrication imperfections. Specifically, for an etching depth of 82.7 nm and asymmetry parameter of 1%, we obtained a record-high *Q* factor of 101,000, which was an orders of magnitude higher than that of typical dielectric metasurfaces governed by symmetry-protected BICs. Additionally, we observed sub-picometer peak wavelength fluctuations in an aqueous solution, demonstrating an improved limit of detection in the order of 10^–5^ for changes in the environmental refractive index. Given that qBIC modes can be easily coupled to normally incident light without the need for delicate coupling systems, we believe that our ultrahigh-*Q* metasurfaces offer a promising platform for a wide range of applications requiring strong light–matter coupling including strong coupling, nonlinear frequency conversion, and quantum photonics.

## Supporting Information Available

Additional details about simulation, fabrication, and optical characterization; electric field distributions; calculation of field confinement factors; characterization of fabrication imperfections from the SEM image; possible cause of sidebands in the spectrum; analysis of the measured spectra with the temporal coupled mode theory; *Q*-analysis for metasurfaces with shallower etching depth; original data of the refractometric sensing results; simulation results of refractometric sensitivity; direct comparison with fully etched metasurfaces; simulated transmittance spectra for varying geometrical parameters (PDF)
