# Supplementary material for: Low-Contrast BIC Metasurfaces with Quality Factors Exceeding 100,000
Source: Nano Lett. 2025 Feb 7;25(7):2777–84. doi: 10.1021/acs.nanolett.4c05880 (PMC11849022; doi:10.1021/acs.nanolett.4c05880)
Supplement: Supplementary file 1 — nl4c05880_si_001.pdf [file nl4c05880_si_001.pdf]

Supporting Information for

Low-Contrast BIC Metasurfaces with Quality Factors Exceeding 100,000

Keisuke Watanabe[1]*, Tadaaki Nagao[1,2], and Masanobu Iwanaga[3]

1.* International Center for Materials Nanoarchitectonics (MANA), National Institute for Materials Science (NIMS), 1-1 Namiki, Tsukuba, Ibaraki 305-0044, Japan.
2. Department of Condensed Matter Physics, Graduate School of Science, Hokkaido University, Kita 10, Nishi 8, Kita-ku, Sapporo 060-0810, Japan.
3. Research Center for Electronic and Optical Materials, National Institute for Materials Science (NIMS), 1-1 Namiki, Tsukuba, Ibaraki 305-0044, Japan.

* Email: watanabe.keisuke@nims.go.jp

S1. Materials and methods

Simulations: Transmittance spectra and electromagnetic mode profiles were simulated using a commercial FDTD solver (Ansys Lumerical). Radiative Q factors were calculated using the FEM (COMSOL Multiphysics) and determined using equation $Q = \mathrm{Re}(f)/2\mathrm{Im}(f)$, where f is the complex eigenfrequency. In both simulations, perfectly matched layers were applied along the z axis, while Bloch (periodic) boundary conditions were used along the x and y axes for the FDTD (FEM).

Fabrication: To fabricate low-contrast silicon metasurfaces, SOI wafers with a 400 nm top silicon layer and a 2000 nm BOX layer were used. The fabrication process is schematically shown in Figure S1. The SOI wafers were initially cleaned in an ultrasonic bath with acetone and IPA, followed by O_2 plasma treatment (AQ-500, Samco). A 100 nm-thick positive resist (ZEP-520A, Zeon Chemicals) diluted 1:1 in anisole was spin-coated onto the SOI wafers and prebaked at 180°C for 3 min on a hotplate. A nanopattern with a sufficiently large dimension of 100 × 100 µm was defined via electron beam lithography (ELS-BODEN, Elionix) at an acceleration voltage of 100 kV. After patterning, the sample was developed in xylene for 1 min and rinsed in IPA for 30 s. The nanopatterns were then transferred into the silicon layer using a Bosch process via dry etching (MUC-21 ASE-SRE, Sumitomo Precision Products) based on SF_6 and C_4H_8 gases. The silicon etching depth was controlled by adjusting the number of etching and passivation cycles in the Bosch process. The etching rate was measured using a spectroscopic ellipsometer (M-2000, J.A.Woollam). Any remaining resist was finally removed by water vapor plasma treatment (AQ-500, Samco).

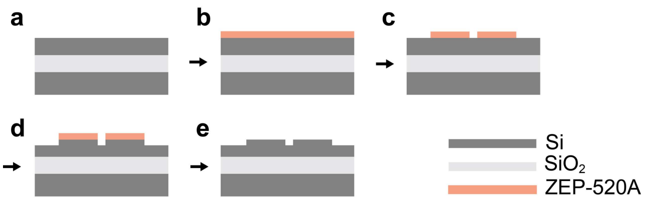

Figure S1. Fabrication process of low-contrast BIC metasurfaces. (a) SOI wafer. (b) Resist coating. (c) Electron beam lithography. (d) Dry etching using the BOSCH process. (e) Resist removal.

Characterization: The transmittance spectra were characterized using a custom-built setup (Figure S2). A tunable single-mode laser (TSL-510, santec) light with x-polarization was directed vertically onto the metasurface through a 10× objective (M Plan Apo NIR, NA = 0.26, Mitsutoyo), and transmitted light was detected by an InGaAs photodiode through a 5× objective (M Plan Apo NIR, NA = 0.14, Mitsutoyo). Background noise was eliminated using a cross-polarized configuration with two polarizers. The photodiode signal was acquired using a data acquisition (DAQ) device (USB-6212, NI) synchronized with the tunable laser (sweep speed of 1 nm/s) and recorded via a LabVIEW (NI) program at a 10 kHz sampling rate. Before measuring the spectra, the native oxide on the metasufaces was removed using O_2 plasma treatment for 5 min (AQ-500, Samco). For refractometric sensing, the fabricated metasurfaces were integrated into a custom-made PDMS microfluidic channel (Micro TAS Engineering), and the liquid solution was exchanged using a peristaltic pump (Takasago Fluidic Systems) after the wavelength drift had stabilized. The refractive index of the solution was pre-measured using a refractometer (PAL-RI, Atago) and adjusted by mixing deuterium oxide D_2O (151882, Sigma-Aldrich) with IPA. During real-time measurements, the resonance peak wavelengths were determined by fitting the transmittance spectra with Fano profiles.

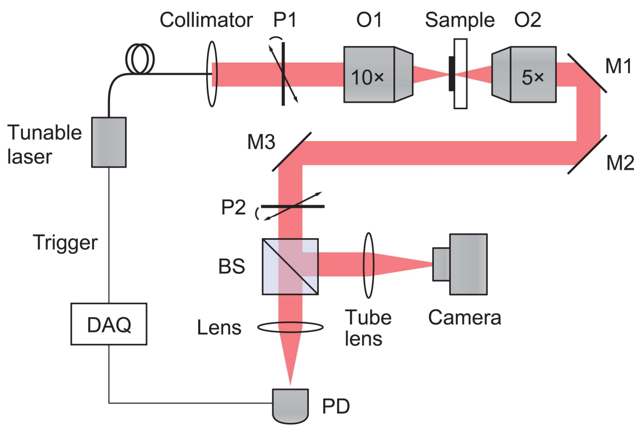

Figure S2. Experimental setup for transmittance measurement. P1 and P2, polarizers; O1 and O2, objectives; M1, M2, and M3, mirrors; BS, beam splitter; PD, photo detector.

S2. Electric field distributions

Figure S3 shows the classifications and mode profiles for the four modes discussed in the main text. The cross-sectional E_x profiles are shown for the qBIC1, qBIC2, guided-mode resonance (GMR), and qBIC3 modes on the *xz* plane (at the center of the lower rod) and *yz* plane (at the center of the unit cell). The qBIC2 mode exhibits small mode localization into surrounding air outside the silicon on both the *xz* and *yz* planes, which can be attributed to the high *Q* factor of qBIC2.

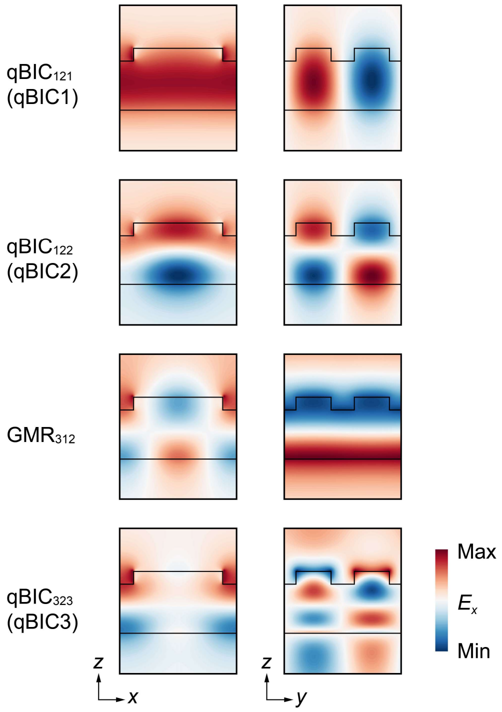

Figure S3. Cross-sectional E_x distributions for qBIC1, qBIC2, GMR, and qBIC3 modes supported by metasurfaces with d = 82.7 nm and α = 5%.

S3. Calculation of field confinement factors

We calculated the field confinement factors in air f_{air} and silicon f_{Si} for all three modes. These confinement factors are defined as the ratio of the electric field energy in air or silicon to the total energy:[1]

$$f_{\mathrm{air}} = \frac{\int_{\mathrm{air}} \varepsilon |E|^2 dV}{\int_{\mathrm{total}} \varepsilon |E|^2 dV}, \quad f_{\mathrm{Si}} = \frac{\int_{\mathrm{Si}} \varepsilon |E|^2 dV}{\int_{\mathrm{total}} \varepsilon |E|^2 dV},$$

where ε represents the dielectric constant of the given material. Table S1 shows the calculated f_{air} and f_{Si} for the three modes using the FDTD method. Seemingly, the qBIC1 mode achieves a higher theoretical *Q* factor considering the highest f_{Si}, but the qBIC1 mode arises in the longer wavelength regime around 2.2 μm. This necessitates the smaller feature sizes to bring the resonance wavelength closer to 1.55 μm, making it more susceptible to fabrication imperfections. Consequently, the experimental *Q* factors for the qBIC1 mode can decrease, as confirmed by our initial measurements. While the qBIC3 mode is spectrally close to the qBIC2 mode, its smaller f_{Si} is not conductive to achieving higher *Q* factors. Therefore, we can conclude that the qBIC2 mode is the most suitable choice for the ultrahigh-*Q* factors, balancing a sufficiently large f_{Si} and a practical feature size.

Table S1. Field confinement factors in air f_{air} and silicon f_{Si} for the three modes (α = 5%).

	qBIC1	qBIC2	qBIC3
f_{air}	0.011	0.021	0.032
f_{Si}	0.967	0.935	0.894

S4. Characterization of fabrication imperfections from the SEM image

We performed quantitative analysis of SEM images to characterize fabrication imperfections, including surface roughness. First, the SEM image shown in Figure S4a was imported into Image J and pre-processed using Gaussian Blur filter. Then, the nanopattern edges were detected and binarized by setting a threshold based on the gradient intensity of each pixel. Each enclosed particle was then labeled (Figure S4b), and the area of each was calculated for N = 99 rods. Here, particles at the image boundaries were excluded. Figure S4c shows the histogram and the fitted Gaussian distribution, yielding a mean $\mu = 1.78 \times 10^5$ nm^2 and a standard deviation $\sigma = 2.07 \times 10^3$ nm^2. The coefficient of variation (CV), defined as σ/μ, was calculated to be 1.1%. This result suggests that radiative qBIC modes can be experimentally observed when the asymmetry parameter α exceeds this value. In reality, however, we observed qBIC modes even at α = 0.5% as indicated in the main text. We think that this discrepancy arises from overestimation of rod areas due to the edge detection algorithm, which includes both surface roughness and adhesions bound to the surface. This explanation is supported by the histogram bins, which is skewed to the right of the mean.

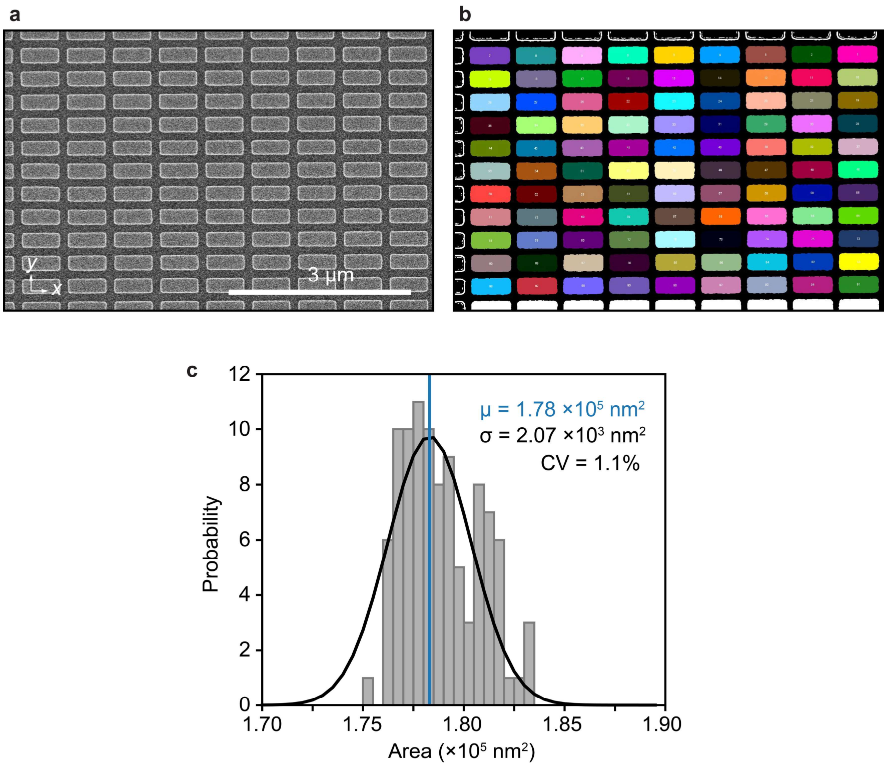

Figure S4. (a),(b) SEM image in the xy-plane and labeled rods in different colors (N = 99) for a low-contrast BIC metasuface (α = 0%, d = 82.7 nm). (c) Histogram of rod areas. The black curve represents the fitted Gaussian distribution, and the vertical blue line is the mean value.

S5. Possible cause of sidebands in the spectrum

Upon investigation of the possible cause of the sidebands observed in the spectra, we found that the in-plane focal position (spot size ~30 µm) of the 100 µm × 100 µm metasurfaces slightly affect the resonance wavelengths. In other words, the observed spectra likely represent a superposition of in-plane resonance wavelength distributions, which may account for the unwanted sidebands. Figure S5 shows a zoomed-in spectrum from Figure 2c for α = 2%, indicating that the sidebands are not entirely random. One plausible explanation for this observation is periodic size variations arising from the writing order with regularity during EB lithography.

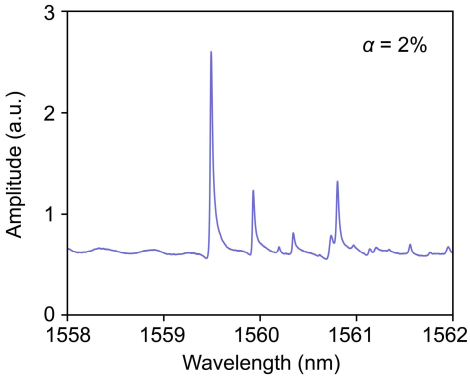

Figure S5. An example of unintended sidebands appeared in the transmittance spectrum for the low-contrast BIC metasuface (α = 2%, d = 82.7 nm).

S6. Analysis of the measured spectra with the temporal coupled mode theory

For more detailed theoretical treatment of Q factors, the experimental Q and Q_{scat} were extracted using temporal coupled mode theory (TCMT). Specifically, we employed an equation for a transmittance system with a single resonator model coupled to two ports:[3,4]

$$T(\lambda_0,\lambda)=\left|t-\frac{Q_r^{-1}}{2i\left[(\lambda-\lambda_0)/\lambda_0\right]+Q_r^{-1}+Q_{scat}^{-1}}(r+t)\right|^2,$$

where λ_0 is the resonance peak wavelength, and Q_r and Q_{scat} are the radiative and scattering Q factors, respectively. t and r are the complex transmission and reflection coefficients, respectively. This formulation explains the Fano lineshapes observed in qBIC modes. Figure S6a shows a representative fitting result, illustrating the extraction of the Q and Q_{scat} while Q_r being the simulated Q factors. We repeated the spectral fitting for different α and d, as shown in Figure S6b. The extracted Q_{scat} remained roughly constant but exhibited a weak decrease for small α and d. This trend can explain the imperfect fitting of experimental Q factors in Figure 3, particularly when d is small. The fluctuations in the extracted Q_{scat} may be attributed to variations in the quality of the nanofabricated metasurfaces and their background noise in the transmittance, which influenced the TCMT fitting conditions. Nevertheless, it remains reasonable to assume that Q_{scat} is shared across all α, thereby simplifying the discussion throughout the paper.

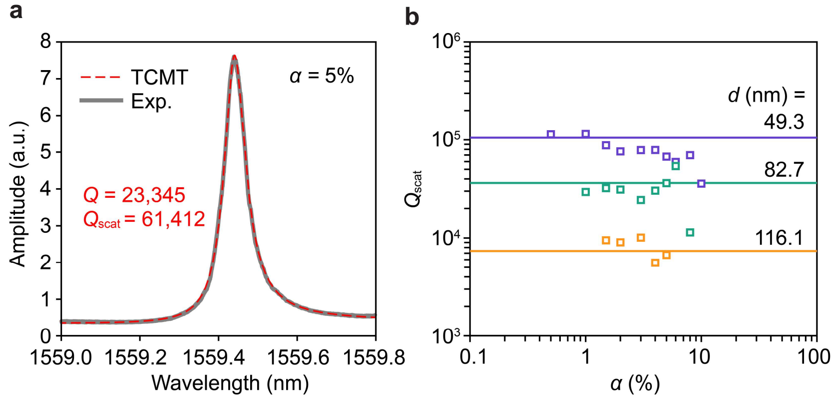

Figure S6. (a) Representative spectrum (gray line) and the corresponding TCMT-based fitting result (red dashed line) for the BIC metasurface (α = 5%, d = 82.7 nm). (b) Comparison of Q_{scat} obtained from Q-analysis presented in Figure 3 (solid horizontal lines) and those derived from TCMT-based fitting (open squares) for various etching depths d.

S7. *Q*-analysis for metasurfaces with shallower etching depth

In the main text, we mention that the experimental *Q* factors did not notably increase for etching depth *d* below 50 nm. To clarify this, Figure S7a,b shows the *Q*-analysis results for the metasurfaces with *d* = 49.3 nm and compares them to those for *d* = 82.7 nm. As expected, the radiative *Q* factor Q_r increases for *d* = 49.3 nm. However, the experimental *Q* factors were comparable to those for *d* = 82.7 nm, particularly when *α* was small. This result can be explained by the scattering loss component (Q_{scat}) reaching a limit of approximately 10^5, as shown in Figure S7c.

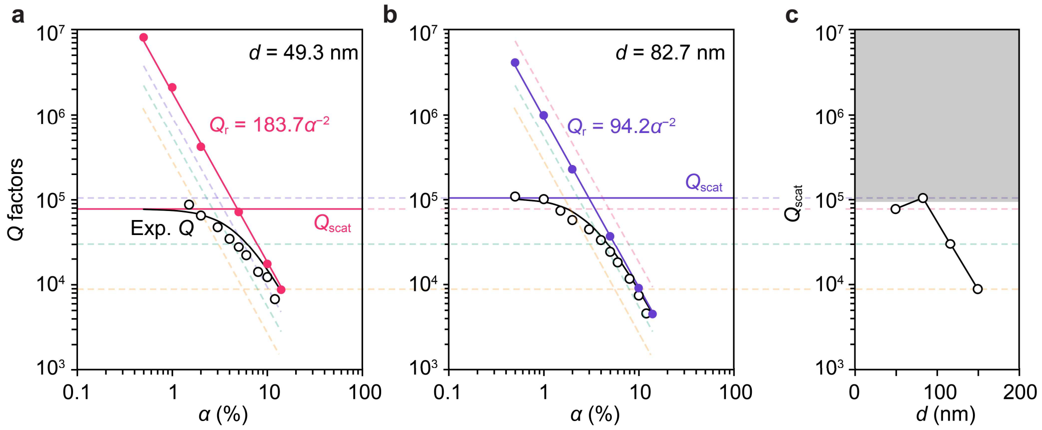

Figure S7. Comparison of *Q* factors for (a) *d* = 49.3 nm and (b) *d* = 82.7 nm. (c) Extracted Q_{scat} as a function of etching depth *d*.

S8. Original data of the refractometric sensing results

Figure S8 shows the real-time refractometric sensing results measured twice (trial 1 and trial 2) for different etching depths *d*. The observed redshift in the elevated baseline is likely due to incomplete rinsing with D_2O. Conversely, a blueshift was occasionally observed following the injection of the solution, particularly when the refractive index change Δn was large. The magnitude of the blueshift appeared random and, in some cases, exceeded the baseline shift observed before the refractive index of the solution was changed after rinsing with D_2O. This suggests that the cause of the blueshift is not straightforward. Given that we used isopropyl alcohol (IPA)/D_2O mixtures, we hypothesize that the blueshift might be caused by a reduction in the surrounding refractive index due to a "surface cleaning" effect on the metasurfaces. This blueshift was more pronounced when the refractive index change was large, indicating that the alcohol-induced cleaning effect became stronger as the IPA concentration increased (IPA/D_2O ratio ~14.2% when Δn = 0.01).

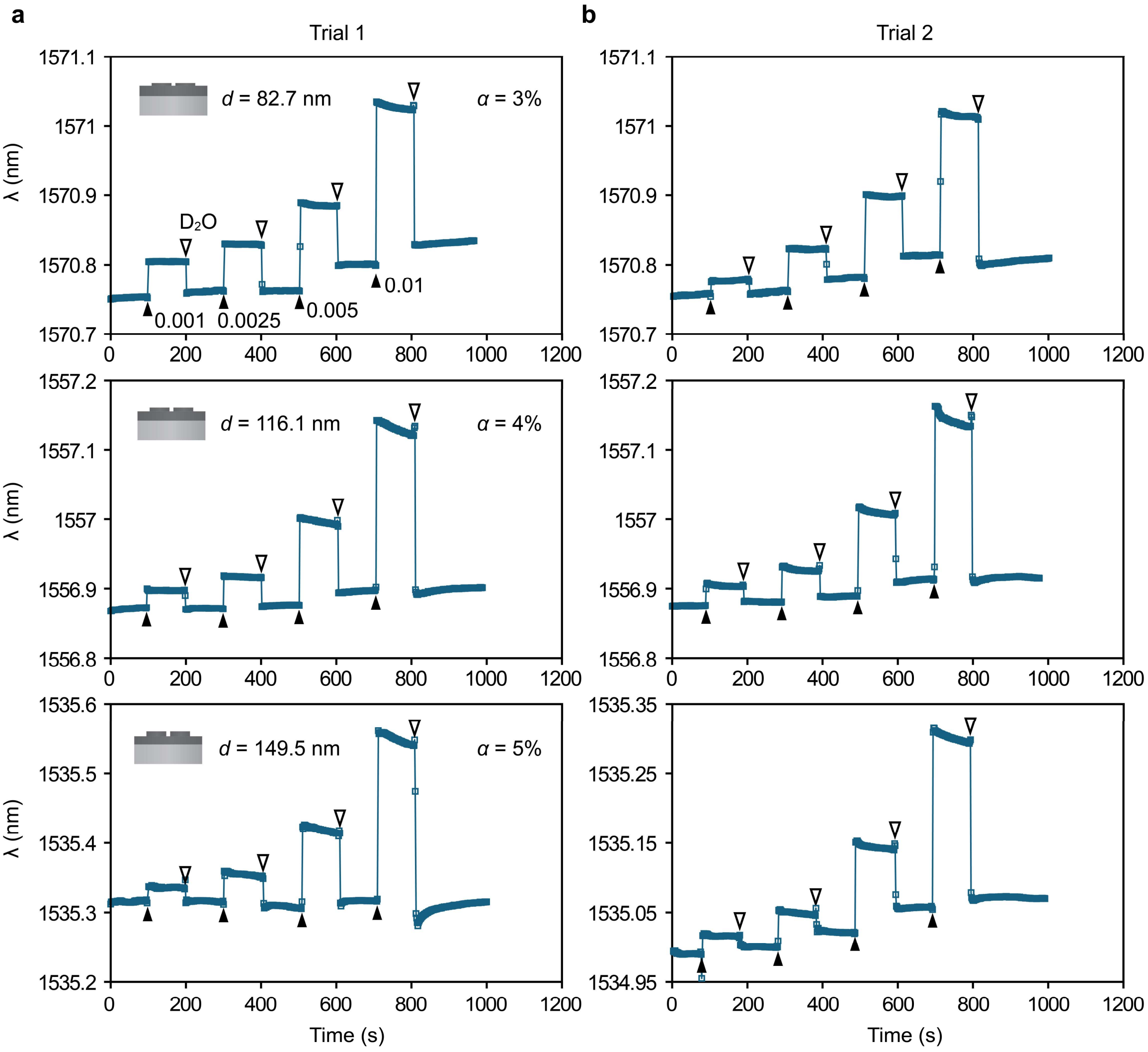

Figure S8. Refractometric sensing results for low-contrast BIC metasurfaces with varying etching depths *d*. Measurements were performed twice, shown in (a) for the first trial (b) for the second trial. D_2O solutions with different refractive indices were introduced sequentially, with refractive index variations of Δn = 0.001, 0.0025, 0.005, and 0.01 (marked by filled triangles). After each step, the metasurfaces were rinsed with D_2O (open triangles).

S9. Simulation results of refractometric sensitivity

The refractive index sensitivity of low-contrast BIC metasurfaces for different α and d was calculated using the FEM by analyzing the shift in eigenfrequencies owing to changes in the environmental refractive index. We confirmed that S increases slightly as d increases (Figure S9), which can be attributed to the reduction in the mode confinement inside the silicon layer. Although the experimental S (Figure 4b) was lower than the calculated values, the difference in S between different d was consistent with the simulation results.

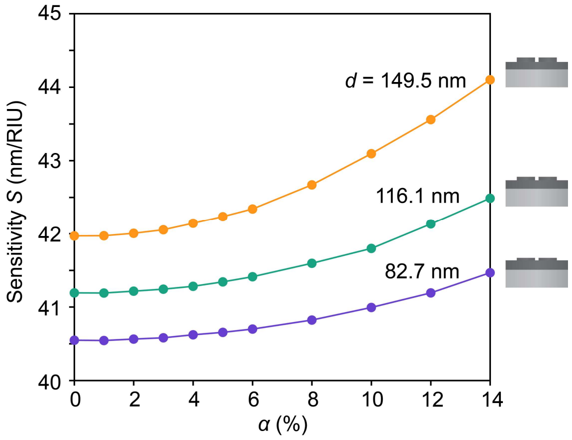

Figure S9. Calculated refractometric sensitivity S as a function of asymmetry α for different d.

S10. Direct comparison with fully-etched metasurfaces

Table S2 shows a comparison of the sensing performance between fully-etched and shallow-etched metasurfaces near the critical coupling conditions. Both metasurfaces share similar structural parameters, differing only in the thickness of the silicon layers *D* and the etching depths *d*. Notably, the fully-etched metasurface exhibits a larger confinement factor in water f_{water}, resulting in a higher refractometric sensitivity *S*. In contrast, the shallow-etched metasurface has a f_{water} approximately ten times smaller. Consequently, its *S* was also reduced by a factor of ten. However, the shallow-etched metasurface demonstrated a *Q* factor approximately ten times larger, attributed to the reduced scattering loss caused by fabrication imperfections. These results are consistent with the general trade-off between the *Q* factor and sensitivity. While the large FOM for the shallow-etched metasurface benefited from the large *Q* factors, the LOD for both geometries were almost the same because their δλ and *S* varied in almost the same proportion.

Table S2. Comparison of fully-etched and shallow-etched metasurfaces.

	Fully-etched	**Shallow-etched**
	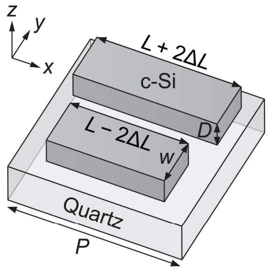	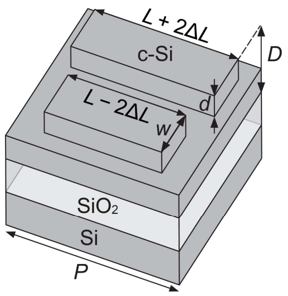
Unit structure	Pair-rod	Shallow pair-rod
Resonance type	SP-BIC	SP-BIC
Material	Silicon on quartz	SOI
(*D, d*)	200 nm, 0 nm	400 nm, 82.7 nm
(*P, L, w*)	790 nm, 595 nm, 230 nm	760 nm, 610.8 nm, 235.5 nm
α	5%	3%
Peak λ	1575 nm	1561 nm
Q_0	2.3	94.2
Exp.*Q* (in air)	433	44,810
Q_{scat} (in air)	818	104,988
S	229 nm/RIU	26.2 nm/RIU
f_{water}	0.30	0.038
δλ	6.4 pm	0.79 pm
FOM	62.8	825
LOD	2.8×10^{-5} RIU	3.0×10^{-5} RIU
Ref.	[4]	**This study**

S11. Simulated transmittance spectra for varying geometrical parameters

We conducted some simulations varying the rod lengths *L*, rod widths *w*, periods *P*, top silicon thickness *D*, and scaling factors *S*. In all simulations, we assumed d/D = 0.2, a condition close to the optimal etching depth *d*. The results are shown in Figure S10. Even with relatively large parameter variations, the qBIC2 modes remain visible in the spectra accompanied by resonance wavelength shifts. However, for sufficiently smaller *w* and larger *P*, the resonance peaks disappear, indicating a drastic reduction in the radiative components of the qBIC2 modes. Compared with variations in *L* and *w*, *P* affects the resonance wavelengths more sensitively (Figure S10a-c). If the silicon thickness *D* of the SOI wafer is increased from 400 nm (real experimental condition) while maintaining d/D = 0.2, the qBIC2 modes remain in the spectra, but an additional qBIC2 mode appears in the longer wavelength region. Cross-sectional E_x distributions for the qBIC2 modes at *D* = 500 nm show that the shorter wavelength peak corresponds to dominant *E*-field confinement near to the bottom of the silicon layer (qBIC2 (↓)), while the longer wavelength peak corresponds to the dominant *E*-field confinement near the rods (qBIC2 (↑)). To further explore the potential for wider wavelength operation, we varied the scaling factor *S* (multiplying all structural parameters except α by *S*), as shown in Figure S11. The wavelength of the qBIC2 mode shifts significantly with *S*, indicating that broad tunability from the near-infrared to mid-infrared regimes is feasible.

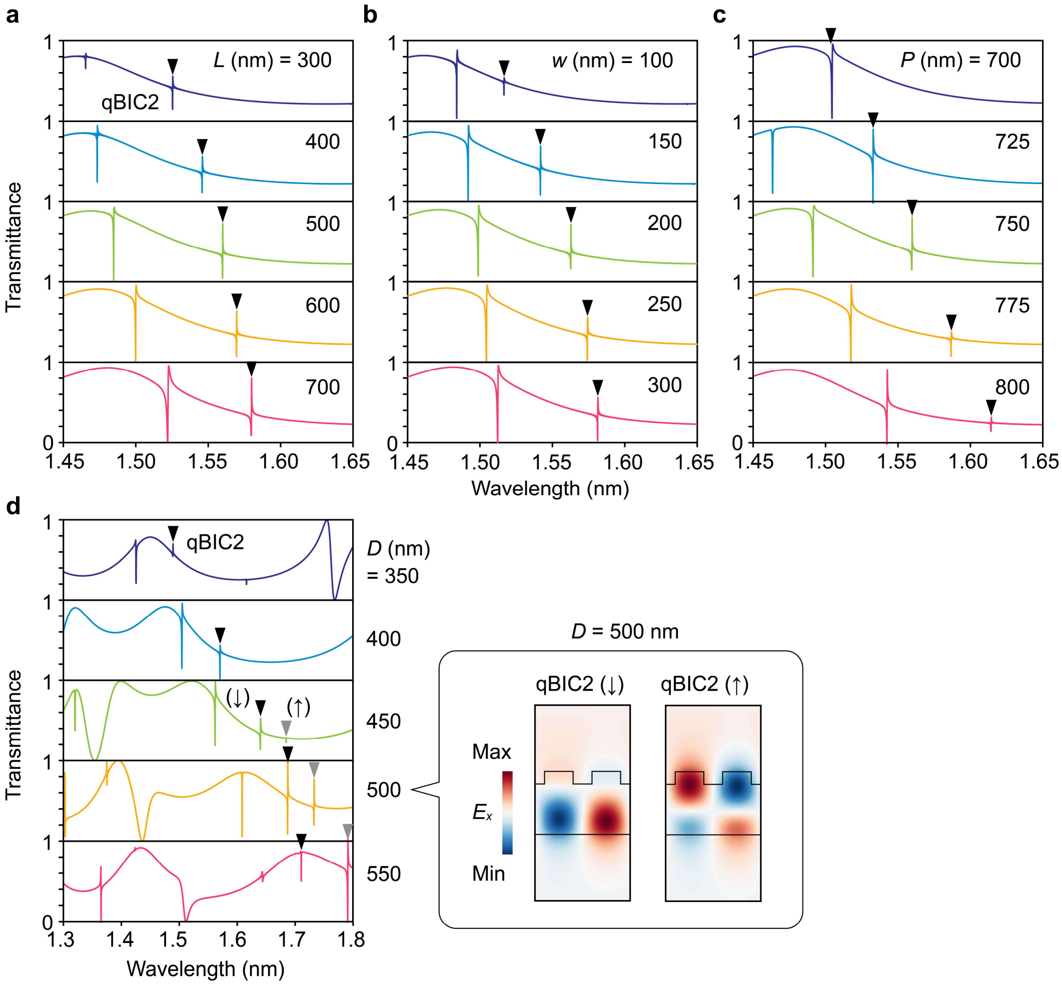

Figure S10. Transmittance spectra of low-contrast BIC metasurfaces (α = 5%, d/D = 0.2) for varying rod lengths *L* (a), rod widths *w* (b), periods *P* (c), and top silicon thickness *D* (d). The triangles indicate the spectral positions of qBIC2 modes. In (d), the inset displays the cross-sectional E_x field distributions for the two types of qBIC2 modes appeared in the spectra at *D* = 500 nm.

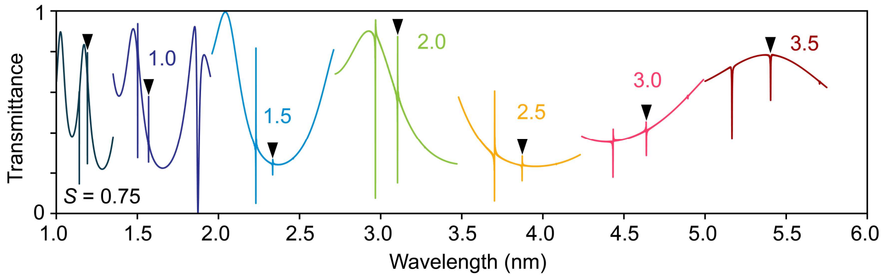

Figure S11. Transmittance spectra of low-contrast BIC metasurfaces (α = 5%, d/D = 0.2) for varying scaling factors S. The triangles indicate the spectral positions of qBIC2 modes.

References

(1) Liu, Y.; Wang, S.; Zhao, D.; Zhou, W.; Sun, Y. High Quality Factor Photonic Crystal Filter at k ≈0 and Its Application for Refractive Index Sensing. *Opt Express* **2017**, 25 (9), 10536.

(2) Fan, S.; Suh, W.; Joannopoulos, J. D. Temporal Coupled-Mode Theory for the Fano Resonance in Optical Resonators. Journal of the *Optical Society of America A* **2003**, 20 (3), 569–572.

(3) Chan, D. L. C.; Celanovic, I.; Joannopoulos, J. D.; Soljačić, M. Emulating One-Dimensional Resonant Q -Matching Behavior in a Two-Dimensional System via Fano Resonances. *Phys Rev A* **2006**, 74 (6), 064901.

(4) Watanabe, K.; Iwanaga, M. Optimum Asymmetry for Nanofabricated Refractometric Sensors at Quasi-Bound States in the Continuum. *Appl Phys Lett* **2024**, 124 (11), 111705.
